# Chemical analysis of additives included in fully formulated oils using high‐performance liquid chromatography–tandem mass spectrometry

**DOI:** 10.1002/rcm.9682

**Published:** 2024-01-16

**Authors:** Vincent Basham, Tom Hancock, John McKendrick, Nathalia Tessarolo, Chrissie Wicking

**Affiliations:** ^1^ Department of Chemistry, School of Chemistry, Food and Pharmacy, Whiteknights Campus University of Reading Berkshire UK; ^2^ BP Technology Centre Berkshire UK

## Abstract

**Rationale:**

Fully formulated oils (FFOs) are chemically complex petrochemical products composed of base oil and additive mixtures that are employed in automotive engines to provide lubrication. In particular, the additive portion of FFOs is often precisely controlled to tailor the resultant formulation to a specific role. Analysis of the additive composition of both used and unused FFOs is therefore of great importance within the petroleum, automotive, and wider engineering industries.

**Methods:**

A simple and rapid reversed‐phase high‐performance liquid chromatography–tandem mass spectrometry method is reported herein for the analysis of a range of additives commonly encountered in FFO samples. Mass spectrometry was performed using an LTQ Orbitrap XL instrument using both positive‐ and negative‐ion electrospray ionization. Tandem mass spectra were acquired in the data‐dependent mode. FFO samples were analysed with minimal sample preparation, limited in this case to simple dilution steps.

**Results:**

The reported method permits analysis of a range of antioxidant, detergent, and antiwear chemistries from various FFO samples in under 10 min. Additionally, it is demonstrated that additive confirmation can be performed and some structural information obtained using the data‐dependent tandem mass spectrometry method. Furthermore, analysis of additives and corresponding degradation products within a used FFO is reported.

**Conclusions:**

The results obtained using the reported methodology are of demonstrable use in numerous industries and applications, and readily return an abundance of information on the additive composition of a range of FFO samples. Anticipated applications of the methodology include but are not limited to quality control, suspected counterfeit analysis, and FFO degradation analysis.

## 1 INTRODUCTION

Fully formulated oils (FFOs) play an invaluable role in lubricating mechanical environments such as automotive engines, where they are responsible for increasing efficiency and extending component lifetimes. Typical compositions of FFOs feature hydrocarbon base oil of either mineral or synthetic origin as the major component, into which speciality chemicals are included to modify the resultant formulation for specific applications and extend the effective lifetime of the product.

Within the engine, FFOs can be exposed to high temperatures and entrained gases. In addition, particulate metals such as iron from mechanical surfaces can also be present within the formulation. Combined, these factors can lead to oxidative degradation of the FFO, the principal mechanism of oil degradation, which decreases FFO performance.^1^, ^2^, ^3^

To counter this, antioxidant additives are nearly always included in formulations and serve to inhibit oxidative depletion of the FFO.^4^, ^5^ Other commonplace additives, such as antifoaming agents, detergents, dispersants, viscosity modifiers, and zinc dialkyldithiophosphates (ZDDPs), are often included to optimize other chemical and physical properties of the formulation.^6^

The resultant formulations produced from additives and base oil are very complex, containing many thousands of distinct chemical compounds. Furthermore, used FFOs can contain a range of additional compounds formed as products of degradation.^7^, ^8^, ^9^ Complex mixtures can be challenging to analysts wishing to understand the chemical composition of such samples. For FFOs in particular a range of analytical techniques have been applied, including infrared spectroscopy, nuclear magnetic resonance spectroscopy, and mass spectrometry (MS), in addition to the standalone or coupled chromatographic techniques liquid chromatography (LC), gas chromatography, and supercritical fluid chromatography.^2^, ^3^, ^4^, ^7^, ^10^, ^11^, ^12^, ^13^, ^14^, ^15^, ^16^, ^17^ Of these, MS utilizing electrospray ionization (ESI) has found value in the analysis of certain FFO additives.^4^, ^18^ However, analysis of samples by ESI‐MS without prior on‐line separation can be hindered by ion suppression, where readily‐ionizing analytes reduce the ionization efficiency of other analytes and in turn diminish the abundance of the latter species in the obtained mass spectra. Often, reversed‐phase high‐performance liquid chromatography (HPLC) coupled to MS can help negate ion suppression, whereby components of a complex mixture are separated according to polarity prior to mass spectrometric analysis. Many modern MS instruments also permit selective dissociation of ions of interest with concomitant mass analysis of the product ions generated in so‐called tandem (MS/MS) analyses. These MS/MS analyses are particularly useful for gaining additional structural information for analytes and can allow confident structure assignment of an ion.

Chromatographic methods reported for the analysis of FFO components often target one particular additive class and require 10 min or more to complete.^4^, ^7^, ^10^, ^14^, ^16^, ^17^ The authors have also reported the development of a dielectric barrier discharge ionization‐mass spectrometric methodology for the analysis of selected additives and base oil components of a model FFO.^19^ This work reports the development and application of a simple, rapid, and versatile HPLC–MS/MS method for the separation and mass analysis of a range of FFO additives of interest across a range of samples, including a model FFO, several commercial products, and a real‐world used FFO. Sample preparation for the reported method is also simple, not requiring extractions or pre‐fractionation.

## 2 MATERIALS AND METHODS

### 2.1 Chemicals and samples

LC–MS‐grade water and methanol were purchased from Fisher Chemicals. LC–MS‐grade toluene was purchased from Honeywell Riedel‐de Haën. FFO products commercially available on the UK market, the used FFO, and FFO components were supplied by BP Castrol. FFO components were prepared in‐house to resemble a finished FFO formulation.

### 2.2 Liquid chromatography–mass spectrometry

A Thermo Scientific Accela HPLC was used for chromatographic separations, with a flow rate of 1 mL/min and column temperature of 40°C. The column used for all analyses was a 50 × 4.6 mm C8 ACE 3 μm (with guard) and the autosampler injection volume was 10 μL. The solvent programme used water and methanol and was as follows: between 0 and 2.5 min, methanol was increased from 75% to 90%, between 2.5 and 2.51 min was then increased to 100% and held until 5.5 min, between 5.5 and 5.51 min the methanol composition was reduced back to 75% and held until 7.5 min. To interface with the mass spectrometer ESI source, the column eluent was split at a ratio of approximately one part to the ESI source and eight parts to waste. All chromatographic analyses were performed in triplicate.

For mass spectrometric analyses, a Thermo Fisher Scientific LTQ Orbitrap XL with an HESI II source was used. For positive ion electrospray analyses, a source voltage of 3.5 kV was used, with capillary and tube lens voltages set to 30 and 80 V, respectively. For negative‐ion electrospray analyses, a source voltage of 3 kV was employed, with capillary and tube lens voltages set to −35 and −110 V, respectively. In both electrospray ion modes, a vaporiser temperature of 250°C and capillary temperature of 275°C was used. Sheath and auxiliary gas flows were set to 45 and 10 arbitrary units, respectively. An automatic gain control target of 10^6^ and maximum inject time of 500 ms were used in both electrospray ion modes with a scan range of *m*/*z* 80–2000 in the Orbitrap mass analyser. A lock mass corresponding to N‐butylbenzenesulfonamide was used in both positive‐ and negative‐ion electrospray modes. For additional structural information, a data‐dependent MS/MS method was employed, where collision‐induced dissociation (CID) was used to generate product ions for subsequent ion trap analysis. A range of activation energies between 30 and 50 arbitrary units were used. In some cases where confident assignment of chemical formulae to product ions analysed in the ion trap was difficult, the product ions were instead analysed in the Orbitrap.

### 2.3 Formulations

To prepare the model FFO formulation in‐house, each additive stock solution was prepared at a concentration of 15 mg of the neat additive per millilitre in toluene, with vortexing to ensure complete dissolution. The antifoam additive stock solution was further diluted by a factor of 100 with toluene. For the model formulation, approximately 1 mL was prepared with an overall concentration of 15 mg FFO per 1 mL, according to the formulation outlined in Table 1. For LC–MS/MS analysis, 10 μL of this formulation was made up to 1 mL using toluene and vortexed to ensure thorough mixing.

**TABLE 1:** Volumes of additives used to prepare the model FFO.^a^

Component	Chemical	Model FFO composition ranges (%)
1	Base oil	80–85
2	Antifoam	0.1–0.5
3	Phenolic antioxidant	0.1–1.0
4	Aminic antioxidant	0.1–1.0
5	Dispersant	5–10
6	Sulfonate detergent 1	0.1–1.0
7	Sulfonate detergent 2	0.1–1.0
8	Phenate detergent	0.1–1.0
9	Viscosity modifier	5–10
10	Secondary ZDDP	0.1–1.0

FFO, fully formulated oil; ZDDP, zinc dialkyldithiophosphate.

^a^ The ranges for each component are reported at the sponsor's request to protect intellectual property. In practice a precise formulation was used where the percentage amount of each component lies within the range reported in Table 1.

### 2.4 Brand samples and used FFO

Selected FFO products available to consumers on the UK market and a used FFO sample were prepared for LC–MS/MS analysis by individually diluting 15 mg of sample in 1 mL of toluene and vortexing. A 10 μL aliquot of the resultant solution was then further diluted to 1 mL using toluene. The properties of the unused FFOs selected for this study are outlined in Table 2. In the case of the used FFO, this was supplied for analysis after use in an engine environment and was of an unspecified mileage.

**TABLE 2:** Properties of the FFOs used in this study.

Product	Brand	SAE grade	API class
1	A	10 W‐50	SL
2	A	15 W‐50	SN
3	A	5 W‐30	SN
4	B	0 W‐30	SN

API, American Petroleum Institute; FFO, fully formulated oil; SAE, Society of Automotive Engineers.

## 3 RESULTS AND DISCUSSION

### 3.1 HPLC‐MS/MS of model FFO

Chromatographic separation of additives within the model FFO was effective and permitted concomitant mass analysis of many additives of interest within the formulation. In particular, highly polar dialkyldithiophosphate (DDP) ligands from the ZDDP additive were seemingly unretained and eluted first, followed by subsequent elution of antioxidants and detergents.

**FIGURE 1:**
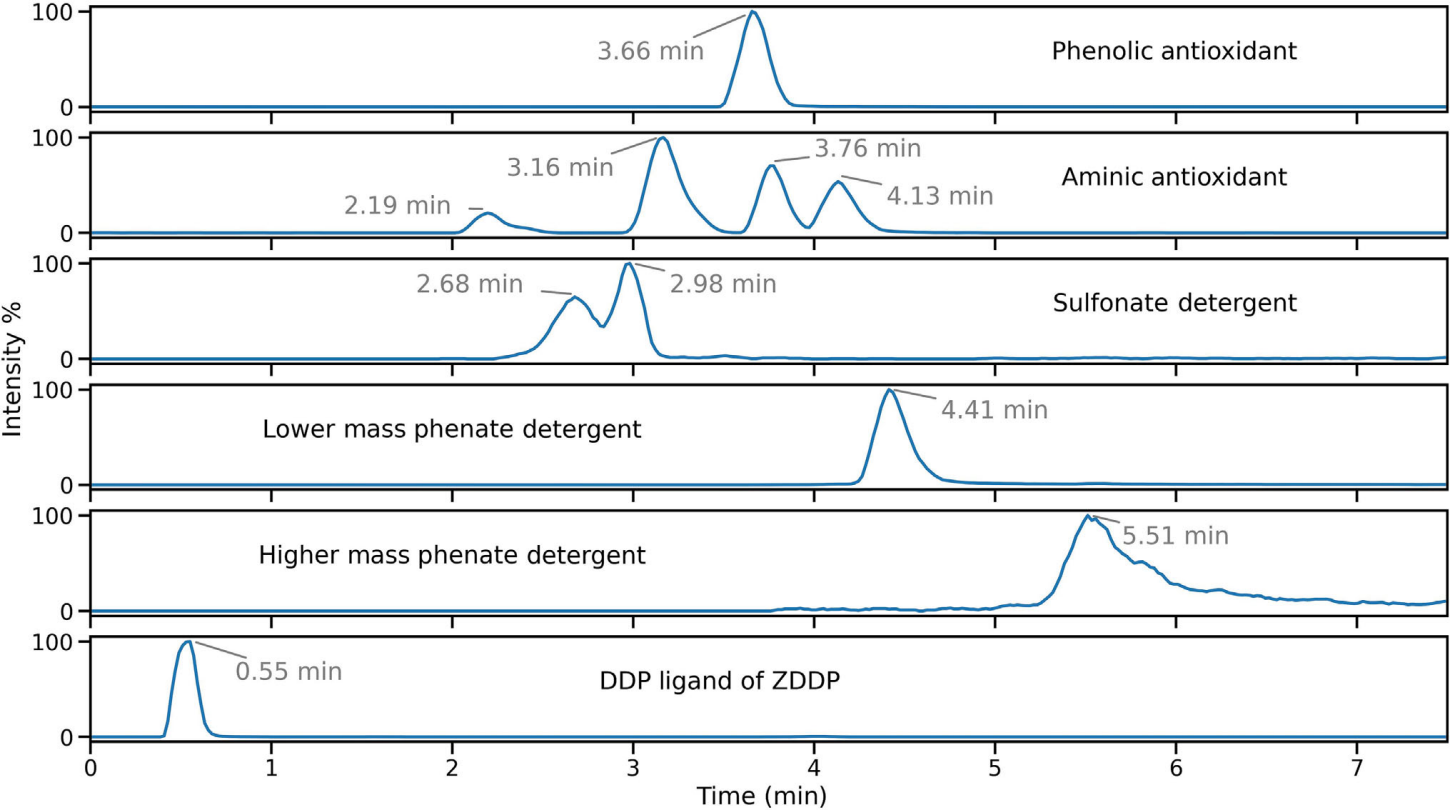
Extracted ion chromatograms of the additive ions detailed in Table 3, representing the elution profiles of additives within the model FFO. DDP, dialkyldithiophosphate; ZDDP, zinc dialkyldithiophosphate. [Color figure can be viewed at wileyonlinelibrary.com]

Analyses in positive‐ion electrospray predominantly yielded information on antioxidants present within formulations. In particular, an intense response of [M + Na]^+^ phenolic antioxidant ions was observed, with concomitant reporting of lower intensity [M + H]^+^ ions. Additionally, a range of substituted diphenylamine derivatives were chromatographically resolved and identified as [M + H]^+^ ions for the aminic antioxidant additive. Specific species derived from ZDDP additives containing a disulphide moiety were also observed as [M + Na]^+^ ions, but the range of ions observed in positive‐ion electrospray for this additive class as a whole was more limited than complementary analyses in negative‐ion electrospray. The origin of the disulphide species formed from ZDDP and observed as [M + Na]^+^ ions is not clear. It is understood these species can be formed from the reaction of ZDDP with alkyl peroxy radicals formed from base oil oxidation within the engine environment, but this pathway is not attributed as the cause for the formation of the disulfide species in the unused and therefore undegraded FFO analysed.^20^ It is also not proposed that these species are formed during the analytical procedure. In negative‐ion electrospray, sulfonate and phenate detergents, as well as DDP ligands of ZDDP complexes, were detected as their corresponding singly charged anions. It is noted that detergents employed in FFOs are manufactured as the metal salt of the corresponding organic acid.^21^ Given that the authors cannot evidence the nature of the metal counterion, no ion types were specified for the detergents which were detected as their conjugate bases. Identification of the phenolic antioxidant is also possible in negative‐ion electrospray from the reporting of [M − H]^−^ ions. Extracted ion chromatograms (EICs) visualized the MS response of ions of interest throughout the chromatography and were used to profile the elution of analytes of interest. EICs for additives in the model FFO, in both electrospray ion modes, and their chemical identities are shown in Figure 1 and Table 3, respectively.

**TABLE 3:** Range of ions identified and assignments from the analysis of the model FFO.

Additive	Structure	Composition ranges	Ion detected	Theoretical mass (Da) of selected ions
Phenolic antioxidant	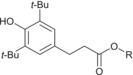	R = C_7_H_15_ to R = C_9_H_19_	[M + Na]^+^	413.3032^a^
			[M + H]^+^	391.3207
			[M − H]^−^	389.3061
Aminic antioxidant	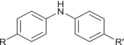	R + R' = C_4_H_10_ to R + R' = C_16_H_34_	[M + H]^+^	226.1590^a^
				282.2216^a^
				338.2842^a^
				394.3468^a^
Sulfonate detergent	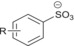	R = C_20_H_41_ to R = C_24_H_49_	As drawn	493.3721^a^
Phenate detergent	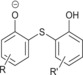	R + R' = C_17_H_36_ to R + R' = C_29_H_60_	As drawn	553.4085^a^
	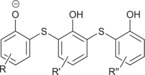	R + R' + R″ = C_33_H_69_ to R + R' + R″ = C_37_H_77_		831.5784^a^
DDP ligand of ZDDP	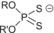	R + R' = C_6_H_14_ to R + R' = C_12_H_26_	As drawn	255.0648^a^
DDP dimer of ZDDP ligands	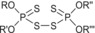	R + R' + R″ + R‴ = C_24_H_52_	[M + Na]^+^	617.2116

R group ranges are reported for clarity. Ions were detected with a mass difference of less than 1 mmu. DDP, dialkyldithiophosphate; FFO, fully formulated oil; ZDDP, zinc dialkyldithiophosphate.

^a^ Ions used for extracted ion chromatograms in Figure 1.

### 3.2 HPLC‐MS/MS of consumer FFO products of unknown composition

In addition to the analysis of a model FFO, several consumer FFO products of unknown composition were analysed (see Table 2). Aminic antioxidants and ZDDP were found to be present in all formulations, but although all products contained at least one detergent, the exact type of this additive varied between formulations. The Brand B product was found to contain both phenate and salicylate detergents, whereas all Brand A products contained only sulfonate detergents. Also of note is the presence of phenolic antioxidant in some products. Both products 3 and 4, from different brands, contained a well‐known phenolic antioxidant employed within lubricant engineering, whereas products 1 and 2, both from Brand A, did not. The differences in composition between products 1–4 are highlighted in Table 4, with EICs reported in Figures S2–S5 of Supporting Information.

**TABLE 4:** Ions identified and assignments from the analysis of a range of commercial products.

Additive	Structure	Product 1	Product 2	Product 3	Product 4
Phenolic antioxidant	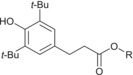	Not detected	Not detected	R = C_7_H_15_ to R = C_9_H_19_	R = C_7_H_15_ to R = C_9_H_19_
Aminic antioxidant	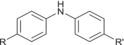	R + R' = C_9_H_20_ to R + R' = C_18_H_38_			
Sulfonate detergent	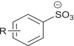	R = C_32_ H_65_ to R = C_48_ H_97_	R = C_32_H_65_ to R = C_48_H_97_	R = C_16_H_33_ to R = C_24_H_49_	Not detected
Phenate detergent	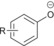	Not detected	Not detected	Not detected	R = C_16_H_33_ to R = C_32_H_65_
Salicylate detergent	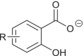	Not detected	Not detected	Not detected	R = C_14_H_29_ to R = C_28_H_57_
DDP ligand of ZDDP	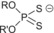	R + R' = C_4_H_10_ to R + R' = C_16_H_34_		R + R' = C_6_H_14_ to R + R' = C_16_H_34_	R + R' = C_3_H_8_ to R + R' = C_12_H_26_
DDP dimer of ZDDP ligands	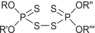	R + R' + R″ + R‴ = C_24_H_52_			

R group ranges are reported for clarity. Ions were detected with a mass difference of less than 1 mmu. DDP, dialkyldithiophosphate; ZDDP, zinc dialkyldithiophosphate.

**FIGURE 2:**
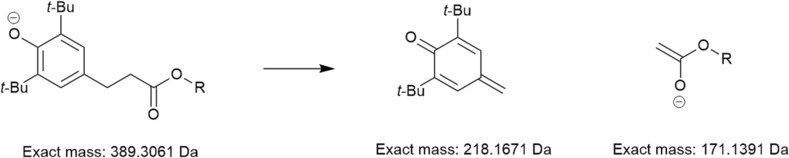
Dissociation observed for phenolic antioxidant in negative‐ion electrospray, leading to a neutral loss of 218 Da and observation of a dominant product ion at *m*/*z* 171. This spectrum was acquired in the Orbitrap mass analyser. *t*‐Bu, *tert*‐butyl.

Many of the analytes yielded characteristic neutral losses on CID, which allowed confident assignment of additive class. In the instance of detergents, sulfonates readily lose SO_2_ with a corresponding decrease in *m*/*z* value of 64, whereas salicylate detergents instead lose CO_2_, characterized by a decrease in *m*/*z* value of 44.^22^ For the salicylate detergent with an R group of C_18_H_37_ this characteristic loss of CO_2_ is particularly useful for distinguishing between the ions derived from a salicylate and phenolic antioxidant of identical exact mass in negative‐ion electrospray, which instead undergoes a charge migration fragmentation process leading to the elimination of the observed charged ester moiety, shown in Figure 2. Such charge migration fragmentations are known to occur over conjugated systems.^23^

DDP ions also show an abundance of informative product ions, where loss of either a single alkyl group or both alkyl groups and an oxygen to yield the OPS_2_^−^ anion is observed, hence indicating the nature of the alkyl substituents.

In aminic antioxidant dissociation, a complex range of ions related to dissociation along the alkyl chains present on the aromatic rings have been reported in the literature and were observed in this work.^5^ Similar alkyl chain dissociation behaviour is observed for phenate detergents, where a characteristic series of methyl unit losses indicates the substituted group is a saturated linear alkane. A summary of the CID energies which yielded the above characteristic losses are detailed in Figure S6 of the Supporting Information.

### 3.3 HPLC‐MS/MS of used FFO of unknown composition

**FIGURE 3:**
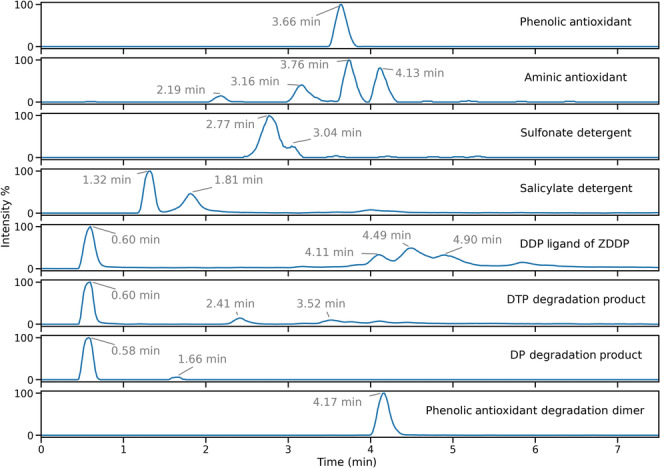
Extracted ion chromatograms of the additive ions detailed in Table 5. DDP, dialkyldithiophosphate; DTP, dialkylthiophosphate; DP, dialkylphosphate; ZDDP, zinc dialkyldithiophosphate. [Color figure can be viewed at wileyonlinelibrary.com]

Across both positive‐ and negative‐ion electrospray analyses, a range of additives were observed within the used FFO sample, in addition to products of degradation. EICs are provided in Figure 3. Principally, a similar variety of undegraded additives was observed when compared to those found in unused products, indicating that in some capacity most additives remain undegraded or are only partially depleted on the timescale of an ordinary oil change interval. In particular, the salicylate and sulfonate detergents were identified within the formulation as their corresponding singly charged anions in negative‐ion electrospray and did not appear to have any related products of degradation. Moreover, phenolic and aminic antioxidant species were observed in positive‐ion electrospray predominantly as their [M + Na]^+^ and [M + H]^+^ ions, respectively, and were of the same composition as those identified in many of the unused consumer products. Of note is the elution profile of seemingly undegraded DDP species, detected as singly charged anions in negative‐ion electrospray, where a second series of elutions was observed after 4.0 min in the chromatography following the initial elution at 0.6 min of largely unretained DDP. Products related to the degradation of the DDP ligands via documented sulphur–oxygen exchange were also observed.^9^ In the first instance, one sulphur–oxygen exchange event yielded a dialkylthiophosphate (DTP) species from DDP on FFO ageing within the engine environment, which may subsequently undergo an additional sulphur–oxygen exchange on continued ageing to form a dialkylphosphate (DP) species. These species were individually observed in these analyses, predominantly as an unretained elution at around 0.6 min, similar to that of the original DDP species from which they are formed. In Figure 3 they are annotated as DTP and DP degradation products accordingly, and they are denoted in Table 5 with superscript letter (b).

**TABLE 5:** Selected ions identified and assignments from the analysis of a real‐world used FFO.

Additive	Structure	Composition ranges	Ion detected	Theoretical mass (Da) of select ions
Phenolic antioxidant	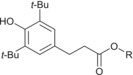	R = C_7_H_15_ to R = C_9_H_19_	[M + Na]^+^	413.3032^a^
			[M + H]^+^	391.3207
			[M − H]^−^	389.3061
Aminic antioxidant	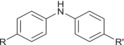	R + R' = C_4_H_10_ to R + R' = C_16_H_34_	[M + H]^+^	226.1590^a^
				282.2216^a^
				338.2842^a^
				394.3468^a^
Salicylate detergent	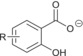	R = C_14_H_29_ to R = C_28_H_57_	As drawn	361.2737^a^
Sulfonate detergent	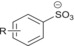	R = C_21_H_43_ to R = C_25_H_51_	As drawn	493.3721^a^
DDP ligand of ZDDP	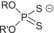	R + R' = C_8_H_18_ to R + R' = C_16_H_34_	As drawn	297.1117^a^
DTP ligand of ZDDP	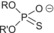	R + R' = C_8_H_18_ to R + R' = C_16_H_34_		281.1335^a^ ^b^
DP ligand of ZDDP	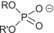	R + R' = C_8_H_18_ to R + R' = C_16_H_34_		265.1563^a^ ^b^
DDP dimer of ZDDP ligands	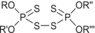	R + R' + R″ + R‴ = C_24_H_52_	[M + Na]^+^	617.2116
Phenolic antioxidant degradation dimer	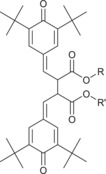	R + R' = C_16_H_34_	[M − H]^−^	773.5715 ^a^ ^b^
			[M + Na]^+^	797.5691^b^

R group ranges are reported for clarity. Ions were detected with a mass difference of less than 1 mmu. DDP, dialkyldithiophosphate; DP, dialkylphosphate; DTP, dialkylthiophosphate; FFO, fully formulated oil; ZDDP, zinc dialkyldithiophosphate.

^a^ Ions used for extracted ion chromatograms in Figure 3.

^b^ Ions identified as degradation products.

All DDP ions observed throughout these analyses yielded visually identical MS/MS spectra via CID as the DDP species observed in products 1 and 2, suggesting they are chemically highly similar, regardless of retention time. This leads to the conclusion that DDP ions observed after 4.0 min in the chromatography are possibly coordinated to other species in solution as a by‐product of FFO ageing within the engine environment, reducing their solvated polarity and hence increasing retention time, and thence within the ESI source prior to mass analysis are released to generate the characteristic singly‐charged anions.

**FIGURE 4:**
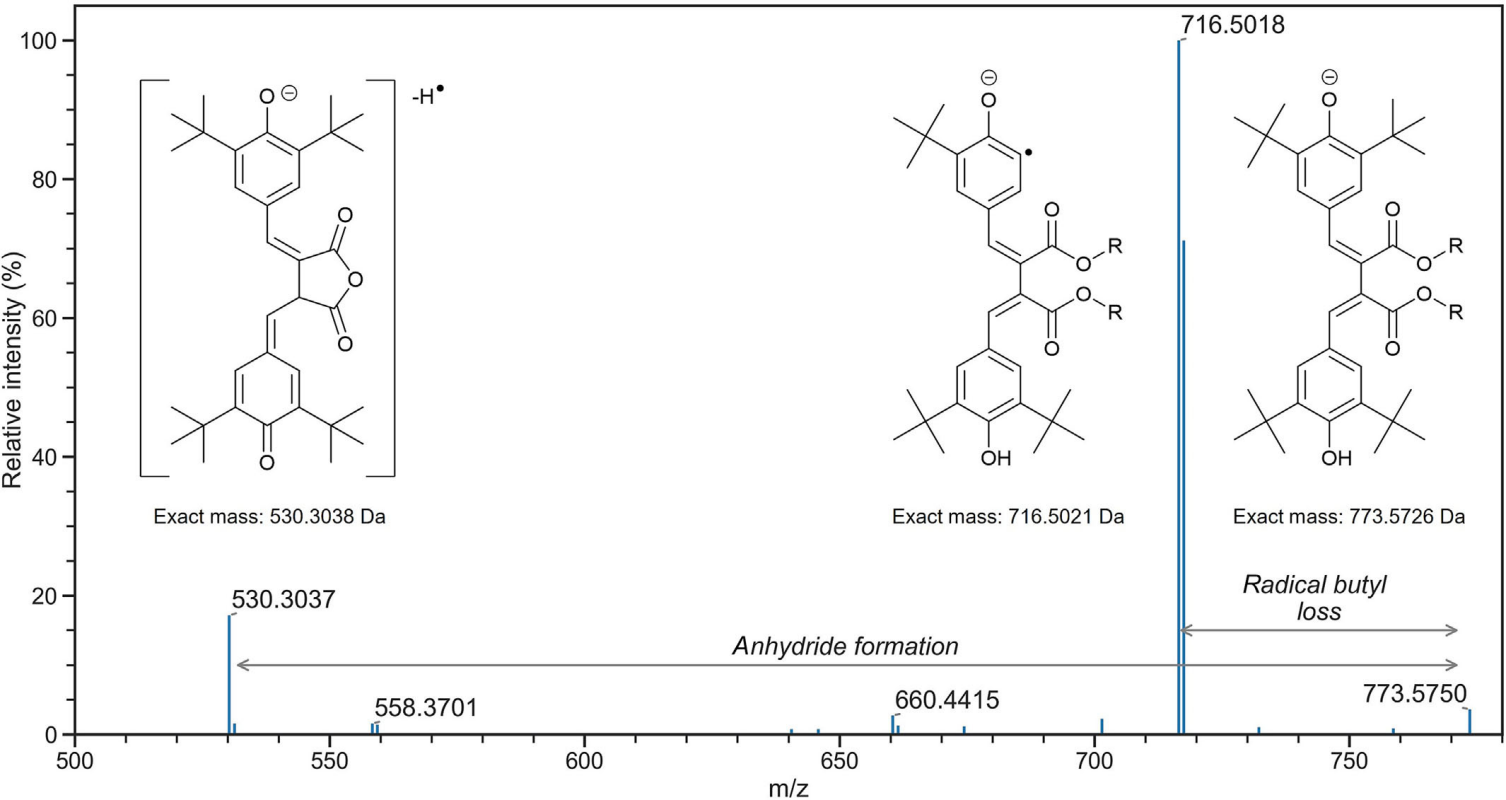
Annotated mass spectrum of product ions generated from CID of the phenolic antioxidant degradation dimer in negative‐ion electrospray. Product ions were analysed using the Orbitrap mass analyser. [Color figure can be viewed at wileyonlinelibrary.com]

It is known that certain additives, in addition to ZDDP, are reactive by design within formulations when in use. As such, they are often consumed to form a range of potential degradation products.^7^, ^8^ Within the used FFO studied, a postulated product of phenolic antioxidant dimerization was assigned the uncharged formula C_50_H_78_O_6_ and observed as [M − H]^−^ ions in negative‐ion electrospray, yielding a highly intense chromatographic peak at 4.17 min, as shown in Figure 3. This species also formed [M + Na]^+^ ions in positive‐ion electrospray, albeit at much lower intensity. Analysis of fragments generated from CID of the [M − H]^−^ ions were analysed using the Orbitrap mass analyser to provide higher confidence in the chemical formulae and therefore identify the two dominant product ions formed, given in Figure 4. The CID energy used was 30 arbitrary units. The neutral loss of 57 Da in the MS/MS spectrum corresponds to the loss of a butyl radical, in keeping with the presence of known *tert*‐butyl substituents in the undegraded phenolic antioxidant. Furthermore, a loss of 243 Da can be attributed to the formation of an anhydride species on dissociation, a pathway known to occur for phthalate ester‐type compounds.^24^ These assignments are given graphically in Figure 4, with structures that represent a best fit to the measured *m*/*z* values and calculated molecular formulae. Diphenylamine‐type aminic antioxidants are also known to undergo extensive reactions as the formulation ages on use, but none of these degradation products were identified in the used FFO in this study.^8^

## 4 CONCLUSIONS

A short and simple HPLC‐MS/MS method was developed for the analysis of FFOs and yielded detailed chemical information on many additives included within these formulations. To the best knowledge of the authors, this is the first time a method of this kind has been reported in the literature.

Application of this method to a range of FFO samples, including a model formulation, consumer FFO products, and a used FFO, demonstrated the versatility of the method and allowed key differences between formulations to be readily and confidently identified. Moreover, MS/MS data for these analytes can provide additional information on additive structure and confidence in additive class assignment.

In addition to the variety of commonplace additives that can be distinguished within unused formulations, a range of analytes related to products of additive degradation were observed in the used FFO sample, principally those originating from phenolic antioxidant and ZDDP species. This information is of particular interest in studies of the complex chemistry of FFO degradation and can allow confident determination of the fate of more labile additives.

## AUTHOR CONTRIBUTIONS

**Vincent Basham:** Formal analysis; investigation; methodology; visualization; writing—original draft; writing ‐ review and editing. **Tom Hancock:** Project administration; resources; supervision; writing—review and editing. **John McKendrick:** Project administration; supervision; writing—review and editing. **Nathalia Tessarolo:** Project administration; resources; supervision; writing—review and editing. **Chrissie Wicking:** Project administration; supervision; resources; writing—review and editing.

### PEER REVIEW

The peer review history for this article is available at https://www.webofscience.com/api/gateway/wos/peer-review/10.1002/rcm.9682.

## Supporting information


**DATA S1.** Supporting Information.

## Data Availability

The data that support the findings are unavailable due to conditions of funding provision.

## References

[1] Blaine S , Savage PE . Reaction pathways in lubricant degradation. 3. Reaction model for n‐hexadecane autoxidation. Ind Eng Chem Res. 1992;31(1):69‐75. doi:10.1021/ie00001a010

[2] Gracia N , Thomas S , Bazin P , Duponchel L , Thibault‐Starzyk F , Lerasle O . Combination of mid‐infrared spectroscopy and chemometric factorization tools to study the oxidation of lubricating base oils. Catal Today. 2010;155(3–4):255‐260. doi:10.1016/j.cattod.2009.11.012

[3] Levermore DM , Josowicz M , Rees JS , Janata J . Headspace analysis of engine oil by gas chromatography/mass spectrometry. Anal Chem. 2001;73(6):1361‐1365. doi:10.1021/ac001157c 11305675

[4] Kassler A , Pittenauer E , Dörr N , Allmaier G . Ultrahigh‐performance liquid chromatography/electrospray ionization linear ion trap Orbitrap mass spectrometry of antioxidants (amines and phenols) applied in lubricant engineering. Rapid Commun Mass Spectrom. 2014;28(1):63‐76. doi:10.1002/rcm.6756 24285391

[5] Kassler A , Pittenauer E , Doerr N , Allmaier G . CID of singly charged antioxidants applied in lubricants by means of a 3D ion trap and a linear ion trap‐Orbitrap mass spectrometer. J Mass Spectrom. 2011;46(6):517‐528. doi:10.1002/jms.1918 21630379

[6] Rudnick LR . Lubricant additives: Chemistry and applications. Second Ed. ( Rudnick LR , ed.). CRC Press; 2009, doi:10.1201/9781420059656.

[7] Kreisberger G , Klampfl CW , Buchberger WW . Determination of antioxidants and corresponding degradation products in fresh and used engine oils. Energy Fuel. 2016;30(9):7638‐7645. doi:10.1021/acs.energyfuels.6b01435

[8] Agarwal S , Singhal S , Singh M , Arora S , Tanwer M . Role of antioxidants in enhancing oxidation stability of biodiesels. ACS Sustain Chem Eng. 2018;6(8):11036‐11049. doi:10.1021/acssuschemeng.8b02523

[9] Dörr N , Agocs A , Besser C , Ristić A , Frauscher M . Engine oils in the field: A comprehensive chemical assessment of engine oil degradation in a passenger car. Tribol Lett. 2019;67(3):68. doi:10.1007/s11249-019-1182-7

[10] Windahl KL , Cardwell TJ . Lability of zinc dialkyldithiophosphates under reversed‐phase high‐performance liquid chromatography conditions. J Chromatogr A. 1997;765(2):181‐186. doi:10.1016/S0021-9673(96)00954-5

[11] Lavison‐Bompard G , Bertoncini F , Thiébaut D , et al. Hypernated supercritical fluid chromatography: potential application for car lubricant analysis. J Chromatogr A. 2012;1270:318‐323. doi:10.1016/j.chroma.2012.10.065 23200024

[12] Owrang F , Mattsson H , Olsson J , Pedersen J . Investigation of oxidation of a mineral and a synthetic engine oil. Thermochim Acta. 2004;413(1–2):241‐248. doi:10.1016/j.tca.2003.09.016

[13] Kupareva A , Mäki‐Arvela P , Grénman H , et al. Chemical characterization of lube oils. Energy Fuel. 2013;27(1):27‐34. doi:10.1021/ef3016816

[14] Snyder SR , Wesdemiotis C . Elucidation of low molecular weight polymers in vehicular engine deposits by multidimensional mass spectrometry. Energy Fuel. 2021;35(2):1691‐1700. doi:10.1021/acs.energyfuels.0c02702

[15] Kiw YM , Adam P , Schaeffer P , Thiébaut B , Boyer C , Obrecht N . Molecular evidence for improved tribological performances of MoDTC induced by methylene‐bis (dithiocarbamates) in engine lubricants. RSC Adv. 2022;12(36):23083‐23090. doi:10.1039/d2ra03036e 36090398 PMC9379776

[16] Hourani N , Muller H , Adam FM , et al. Structural level characterization of base oils using advanced analytical techniques. Energy Fuel. 2015;29(5):2962‐2970. doi:10.1021/acs.energyfuels.5b00038

[17] Lambropoulos N , Cardwell TTJ , Caridi D , Marriott PJP , Candl D , Marriott PJP . Separation of zinc dialkyldithiophosphates in lubricating oil additives by normal‐phase high‐performance liquid chromatography. J Chromatogr A. 1996;749(1–2):87‐94. doi:10.1016/0021-9673(96)00416-5

[18] Dörr N , Brenner J , Ristić A , et al. Correlation between engine oil degradation, tribochemistry, and tribological behavior with focus on ZDDP deterioration. Tribol Lett. 2019;67(2):62. doi:10.1007/s11249-019-1176-5

[19] Basham V , Hancock T , Mckendrick J , Tessarolo N , Wicking C . Detailed chemical analysis of a fully formulated oil using dielectric barrier discharge ionisation–mass spectrometry. Rapid Commun Mass Spectrom. 2022;36(14):e9320. doi:10.1002/RCM.9320 35484791 PMC9286547

[20] McDonald RA . Zinc Dithiophosphates. In: Rudnick LR , ed. Lubricant additives: Chemistry and applications. Second ed. CRC Press; 2009:51‐62. doi:10.1201/9781420059656-c2

[21] Rizvi SQA . Detergents. In: Rudnick LR , ed. Lubricant additives: Chemistry and applications. Second ed. CRC Press; 2009:123‐141. doi:10.1201/9781420059656-c4

[22] Levsen K , Schiebel HM , Terlouw JK , et al. Even‐electron ions: a systematic study of the neutral species lost in the dissociation of quasi‐molecular ions. J Mass Spectrom. 2007;42(8):1024‐1044. doi:10.1002/jms.1234 17605143

[23] Demarque DP , Crotti AEM , Vessecchi R , Ao J , Lopes LC , Lopes NP . Fragmentation reactions using electrospray ionization mass spectrometry: an important tool for the structural elucidation and characterization of synthetic and natural products. Nat Prod Rep. 2016;33(3):367‐524. doi:10.1039/c5np00073d 26673733

[24] Viñas P , Campillo N , Pastor‐Belda M , Oller A , Hernández‐Córdoba M . Determination of phthalate esters in cleaning and personal care products by dispersive liquid‐liquid microextraction and liquid chromatography‐tandem mass spectrometry. J Chromatogr A. 2015;1376:18‐25. doi:10.1016/j.chroma.2014.12.012 25537172

