# Supplementary material for: Chemical analysis of additives included in fully formulated oils using high‐performance liquid chromatography–tandem mass spectrometry
Source: Rapid Commun Mass Spectrom. 2024 Jan 16;38(5):e9682. doi: 10.1002/rcm.9682 (PMC10909512; doi:10.1002/rcm.9682)
Supplement: Supplementary file 1 — DATA S1. Supporting Information. [file RCM-38-e9682-s001.pdf]

Chemical Analysis of Additives Included in Fully Formulated Oils Using High-Performance Liquid Chromatography-Tandem Mass Spectrometry

Supplementary Information

Table S.1

Additive	Ion Type	m/z	Amount on-column (ng)	Signal-to-noise ratio of EIC peak (± standard deviation)
Aminic antioxidant	$[M+H]^+$	226.1590	15.00	16.0 (±1.4)
		282.2216		12.0 (±1.4)
		338.2842		59.7 (±22.2)
		394.3468		41.0 (±36.4)
Phenolic antioxidant	$[M+Na]^+$	413.3032	3.75	248.7 (±35.9)
Phenate detergent	As drawn in Table 3	553.4085	11.25	37.0 (±13.5)
Sulfonate detergent		493.3721	375.00	22.3 (±4.0)
DDP ions of ZDDP		255.0648	37.50	186.7 (±39.1)

Standard deviation values provided in Table S.1 are calculated from 3 repeat measurements.

S.2 – Product 1 EIC

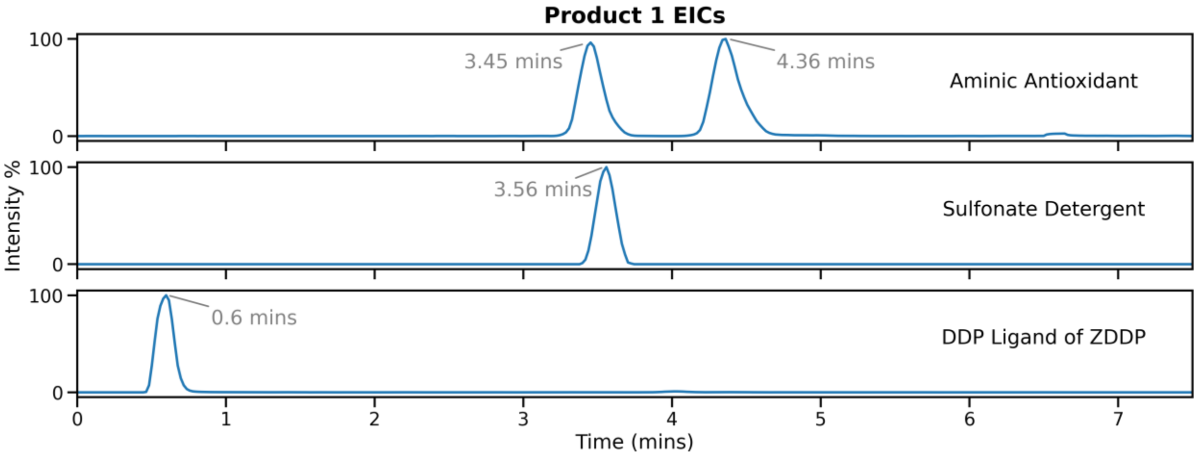

S.3 – Product 2 EIC

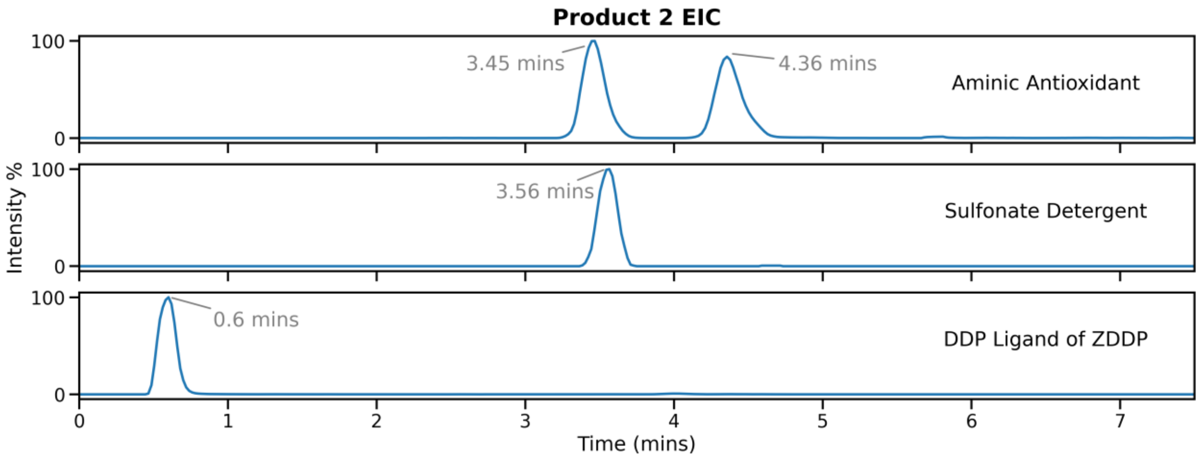

S.4 – Product 3 EIC

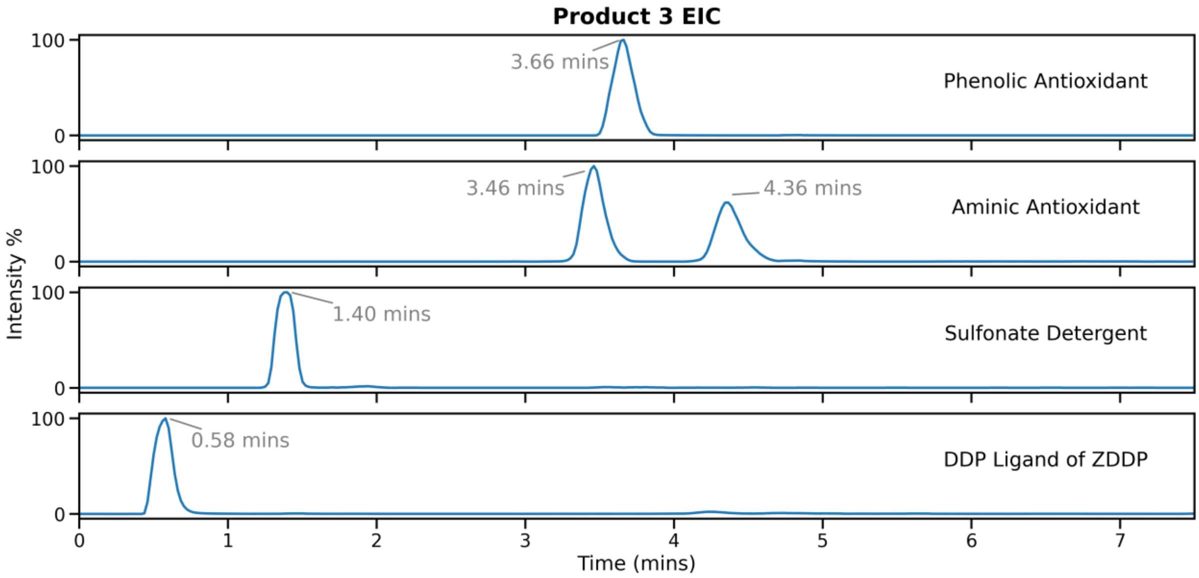

S.5 – Product 4 EIC

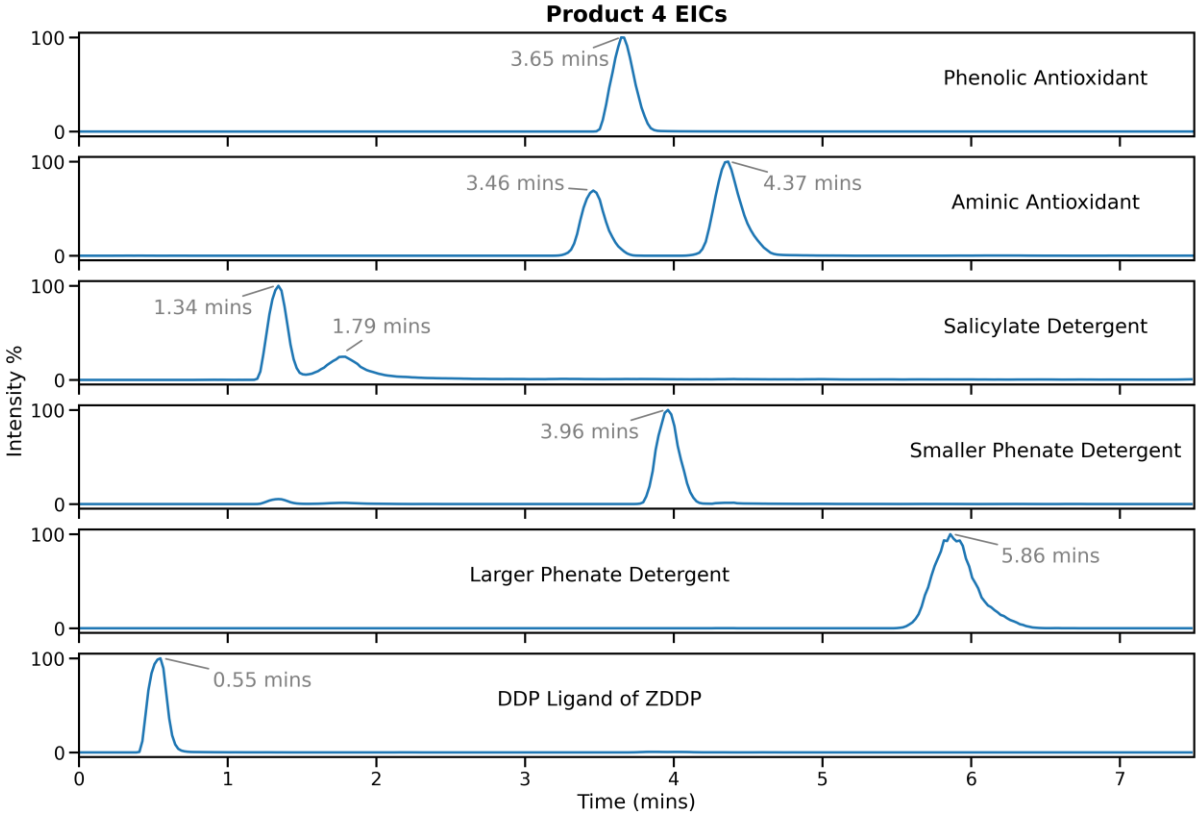

S.6 – CID energies for analytes

Additive	Structure	Ion	Characteristic Neutral Loss (Da, *where applicable*)	Optimum CID Energy (Arb. Units)
Phenolic Antioxidant	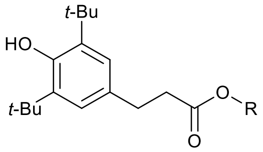	$[M-H]^-$	218 (ester elimination)	30
Phenolic Antioxidant Degradation Dimer	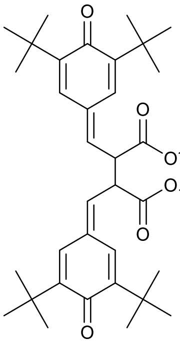	$[M-H]^-$	57 (butyl radical)<br><br>243 (complex, related to anhydride formation)	30
Aminic Antioxidant	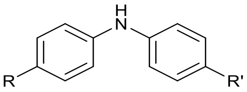	$[M+H]^+$	Complex	30
		$[M-H]^-$	Complex	30
Sulfonate Detergent	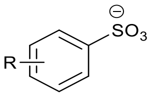	As drawn	64 (SO_2)	35
Phenate Detergent	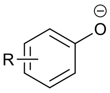	As drawn	Complex	40
Salicylate Detergent	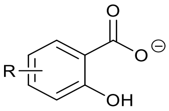	As drawn	44 (CO_2)	30
DDP Ligand of ZDDP	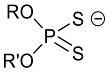	As drawn	Alkyl group, alkyl groups and oxygen	30
DTP Ligand of ZDDP	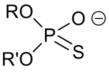	As drawn	Alkyl group, alkyl groups and oxygen	30
DP Ligand of ZDDP	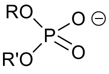	As drawn	Alkyl group, alkyl groups and oxygen	30
